# Crystallographic model quality at a glance

**DOI:** 10.1107/S0907444908044296

**Published:** 2009-02-20

**Authors:** Ludmila Urzhumtseva, Pavel V. Afonine, Paul D. Adams, Alexandre Urzhumtsev

**Affiliations:** a Architecture et Réactivité de l’ARN, Université Louis Pasteur, Institut de Biologie Moléculaire et Cellulaire, CNRS, 15 Rue René Descartes, 67084 Strasbourg, France; b Lawrence Berkeley National Laboratory, One Cyclotron Road, BLDG 64R0121, Berkeley, CA 94720, USA; c Institut de Génétique et de Biologie Moléculaire et Cellulaire, Département de Biologie et de Génomique Structurales, CNRS–ULP–INSERM, 1 Rue Laurent Fries, 67404 Illkirch, France; d Physics Department, Nancy-University, 54506 Vandoeuvre-lès-Nancy, France

**Keywords:** model quality, PDB, validation, refinement, *PHENIX*

## Abstract

A crystallographic macromolecular model is typically characterized by a list of quality criteria, such as *R* factors, deviations from ideal stereochemistry and average *B* factors, which are usually provided as tables in publications or in structural databases. In order to facilitate a quick model-quality evaluation, a graphical representation is proposed. Each key parameter such as *R* factor or bond-length deviation from ‘ideal values’ is shown graphically as a point on a ‘ruler’. These rulers are plotted as a set of lines with the same origin, forming a hub and spokes. Different parts of the rulers are coloured differently to reflect the frequency (red for a low frequency, blue for a high frequency) with which the corresponding values are observed in a reference set of structures determined previously. The points for a given model marked on these lines are connected to form a polygon. A polygon that is strongly compressed or dilated along some axes reveals unusually low or high values of the corresponding characteristics. Polygon vertices in ‘red zones’ indicate parameters which lie outside typical values.

## Abstract 2

The representation of crystallographic model characteristics in the form of a polygon allows the quick comparison of a model with a set of previously solved structures.

## 1. Introduction

Crystallographic macromolecular models possess different types of errors (see, for example, Kleywegt, 2001 ▶ and references therein; Dym *et al.*, 2001 ▶; Brown & Ramaswamy, 2007 ▶; Borman, 2007 ▶; Wlodawer *et al.*, 2008 ▶). The model characteristics that reflect them are usually given either as a list of numbers or in the form of numerous plots and images (see Wodak *et al.*, 2001 ▶) produced, for example, by *PRO­CHECK* (Laskowski *et al.*, 1993 ▶) or *MolProbity* (Davis *et al.*, 2004 ▶). A tool that illustrates model quality in a single image would be helpful.

Each individual parameter such as *R* factor or mean bond-length error may be plotted along a corresponding ‘ruler’ (see, for example, Fig. 5 in Wlodawer *et al.*, 2008 ▶). We suggest arranging these rulers as lines or axes radiating from a common origin. We mark the value of each parameter for a model at a point along these axial rulers. We then join each of these points together with its neighbours to construct a polygon. If one of the characteristics is unusually good (for example, the *R* factor is unusually small), the point along this axis will be closer to the origin. Conversely, for a large *R* factor the point will be far from the origin and the polygon will be expanded along this axis, immediately indicating a deviation from typically observed values. The same image may also present the distribution of each parameter for a set of control models. For example, different parts of axes can be shown in different colours as a function of the frequency of the values. This graphical information answers at a glance common questions such as ‘I’m refining my structure at 2.2 Å resolution and the *R*/*R*_free_ factors are 0.25/0.30; how does my structure compare with other structures refined at the same resolution?’ or similar questions for other model parameters.

## 2. Polygon presentation of model characteristics

### 2.1. Model characteristics

A polygon may be built for any set of model characteristics that are available in PDB files or that can be recomputed given a PDB file and diffraction data files, for example *R* and *R*_free_ factors, deviations from ideal stereochemistry and so on. Reporting only the mean values of the deviations from standard geometry, which are global model characteristics, may be insufficient (Morffew & Moss, 1983 ▶; Urzhumtsev *et al.*, 1989 ▶; Laskowski *et al.*, 1993 ▶); for a more complete estimation of model quality, maximal distortion values (Urzhumtsev, 1992 ▶) should also be communicated. In some ways, maximal deviations can characterize locally different kinds of geometry distortion. Therefore, our ‘default polygon’ includes eight axes for the mean and maximal deviations in the bond lengths, bond angles, di­hedral angles and planarity. Two further default axes show the mean and maximal value of the ADPs (atomic displacement parameters) or their iso­tropic equivalents.

Any of these characteristics can be removed from the polygon, replaced or complemented by other values such as the distortion in chirality, minimal nonbonded distance, number of ordered water molecules per residue, number of outliers in the Ramachandran plot (Ramakrishnan & Ramachandran, 1965 ▶) or the percentage of residues in favourable configurations.

### 2.2. Scaling and colouring

To plot the distribution *f*(*x*) of a characteristic *x*, the interval (*x*_min_, *x*_max_) and its position on the corresponding axis need to be chosen. We preferred to avoid scales that were fixed at standards for geo­metric characteristics (see, for example, Jaskolski *et al.*, 2007 ▶; Stec, 2007 ▶) or *R* factors (Tickle *et al.*, 1998 ▶, 2000 ▶). Instead, we referred to previously solved models.

A straightforward way would be to calculate the mean *x*_mean_ and standard deviation σ_*x*_ of each parameter *x*, define the position of *x*_mean_ for all parameters at the same distance from the origin and plot all *f*(*x*) in the same intervals of σ, for example (*x*_mean_ − 5σ_*x*_, *x*_mean_ + 5*σ*_*x*_), showing them as axes of the same length. With such a choice, the ‘mean model’ polygon would be exactly regular. Outliers can strongly influence the statistics and should be removed in advance. However, even with outliers excluded the distributions for many of the characteristics of the PDB models are multimodal; *x*_mean_ may be between two peaks and may correspond to an unusual value. Therefore, it might be misleading to choose *x*_mean_ as a value for a ‘mean-quality model’. The choice of a standard interval is also inconvenient. Owing to the high diversity of the distributions *f*(*x*), a large part of some intervals may be empty while important information is lost for others.

Another possibility is to take *x*_min_ and *x*_max_ as the minimal and maximal *x* values for the models selected for comparison. Removing outliers makes the definition of *x*_min_ and *x*_max_ insensitive to minor variations in the set of control models. We then plot all *x*_min_ at the same distance from the origin and similarly for *x*_max_. The exceptions are the nonbonded distance and the percentage of residues in the favourable zones of the Ramachandran plot, for which the points for *x*_min_ and *x*_max_ on the axis are flipped.

With this choice, the polygon for the ‘mean-quality model’ is not exactly regular. This imperfection of the current scaling does not cause much inconvenience, particularly because the definition of such a model for multimodal distributions is ambiguous anyway. The important point is that the extremities of the interval correspond to less usual values and, as a consequence, a compressed or dilated polygon indicates an atypical model. (We exclude particular cases in which control models are chosen on purpose to give frequent values at the extremities of the interval; see the next section for a discussion of model selection.) Obviously, more sophisticated scaling schemes may be tried in future.

The axes are coloured according to the frequency with which given values of the parameter are observed in the set of control models. Red corresponds to rare values, green is for ‘usual’ values and blue indicates very frequent zones.

## 3. Models for comparison

When choosing the models for comparison, one may exclude am­biguous models, for example those with a negative difference *R*_free_ − *R* or those that are formally correct but disagree with advanced analysis (see, for example, Jaskolski *et al.*, 2007 ▶). The further choice of control models depends on the questions that are posed. In particular, only models with a particular feature (refinement program, space group, type of experimental data *etc*.) may be retained for comparison.

By default, a model is compared with structures obtained at the same resolution. If the filtered database contains too few such models, the models that are closest in resolution are added from both resolution ends. When working at very high or at low resolution, the resolution limits may be defined explicitly. This option also allows the same set of control models to be chosen for multiple comparisons, in particular the selection of models refined at high resolution (and to low values of the *R* factor) as a high-quality standard. Other selections, for example a similarity in molecular size, may be applied.

## 4. Computer realisation

To illustrate this approach, a Tcl/Tk-based (Ousterhout, 1993 ▶) pro­gram has been written. The model information used to plot the polygon can be taken from any of three different sources: the PDB file header, the output of *phenix.model_vs_data* (a component of *PHENIX*; Adams *et al.*, 2002 ▶) or an internal database of model characteristics recovered from the PDB (see below). Numerous filtering and selection options (discussed in §[Sec sec3]3) are available. The default program parameters are highly customizable.

We used tools from *PHENIX* to extract and collect statistical in­formation from the PDB (Bernstein *et al.*, 1977 ▶; Berman *et al.*, 2000 ▶). Only models with experimental data available were considered and *phenix.cif_as_mtz* was used to extract and convert the experimental data to MTZ format (structure factors, σ values and free *R* flags). This generated a total of 30 448 MTZ files (as of July 2008).

**Figure 1:**
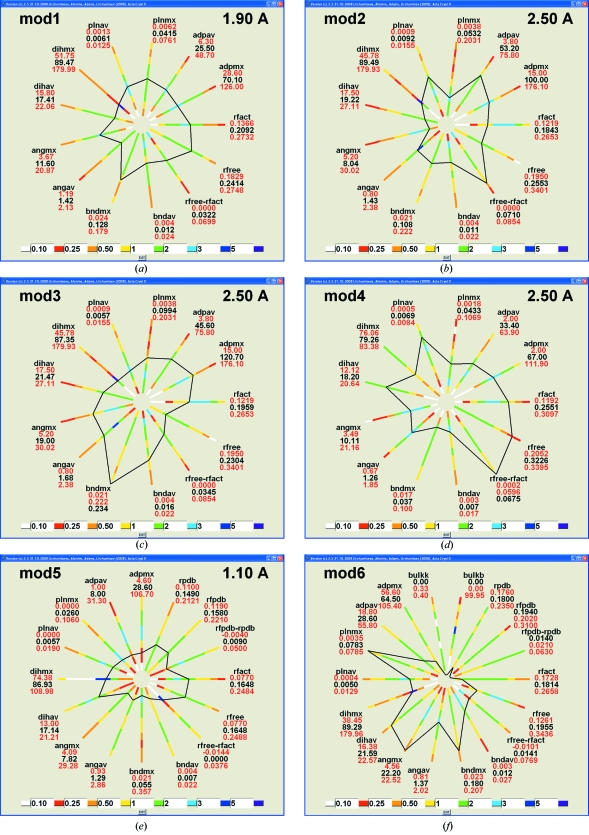
Examples of the polygon presentation of model characteristics. The values bndav, angav, dihav and plnav are the mean deviations from the standard values for bonds, angles, dihedral angles and plane groups, respectively; bndmx, angmx, dihmx and plnmx are their maximal values. adpav and adpmx are the mean and maximal values of the atomic displacement parameters or their isotropic equivalents. Axes are coloured accordingly to the frequency of the model characteristics for the selected set of PDB models with a particular resolution (given in the upper right corner). The values of the given frequency (for example, green for a frequency between 1 and 2) show how much higher or lower it is than the frequency for the uniform distribution. See the text for details and comments.

We used *phenix.model_vs_data* to homogenously compute model and data statistics such as *R* factors or stereochemical deviations (*PHENIX* uses the CCP4 Monomer Library; Vagin *et al.*, 2004 ▶). The original values of the *R* factors from the headers of the PDB files can be displayed at additional axes of the polygon (see, for example, the axes rpdb and rfpdb in Figs. 1 ▶ *e* and 1 ▶ *f*). The models with a large difference between reported and calculated *R* factors can be filtered out by request.

## 5. Examples of the polygon representation

Fig. 1 ▶ shows polygon representations of several models taken from the PDB. The actual PDB codes were substituted by the artificial codes mod1–mod6. The resolution displayed corresponds to that of the data set in the MTZ file. The coloured axes show the frequency of corresponding values, with the numerical limits indicated in red. The characteristics for the input model are given in black. The values are given in conventional units: relative values for *R* factors, Å for bond-length deviations, degrees for angles, Å^2^ for ADPs *etc*.

Mod1 (Fig. 1 ▶ *a*) shows characteristics typical of other models at this resolution. The characteristics are close to the centres of the distributions and the polygon is approximately radially symmetric.

Mod2 (Fig. 1 ▶ *b*) also has typical values for the geometric characteristics. However, its *R* factor is lower and Δ*R* (*R*_free_ − *R*) is larger than highly frequent values, suggesting some degree of overfitting of the data.

Mod3 (Fig. 1 ▶ *c*) shows a high maximal deviation of bond lengths (while the mean value is close to typical values), indicating the pre­sence of small number of local model imperfections. It also shows a similar trend with bond angles and planarity.

Mod4 (Fig. 1 ▶ *d*) shows a good agreement for geometry values, but has high *R* and *R*_free_ factors and a high Δ*R*. For illustration, only 1% of outliers with very small or very large values of the model characteristics were rejected automatically instead of the 10% that were rejected for the other figures. In contrast, here we applied an ‘explicit filtering’ that excluded control models with Δ*R* < 0.0001, with a maximal deviation in dihedral angles larger than 150° and with a maximal deviation in bond length larger than 0.10 Å.

Mod5 (Fig. 1 ▶ *e*), which was refined at a high resolution, has most of the geometry parameters equal to or smaller than typical values. The small mean and maximal values of the isotropic equivalent of the ADPs suggest that the structure may be highly ordered. The *R* factors reported in the PDB header (shown as three additional axes) are low. However, the value of zero for the calculated Δ*R* signifies that for this model the actual test set of structure factors is not available in the PDB. This prevents calculation of the *R*_free_ factor using the deposited data. The blue colour of the corresponding interval, which stands for very typical values, indicates a high percentage of PDB models with this feature.

The obviously irregular polygon for mod6 (Fig. 1 ▶ *f*) corresponds to a model with serious problems. The resolution has been removed from this figure on purpose.

## 6. Conclusions

The presentation of a set of commonly used model characteristics in one image allows an easy assessment of model quality and comparison with a set of control models. The approach does not suggest a new measure of the model quality, but provides a convenient way to evaluate it at a glance. Obviously, a similar technique can be used to analyze other types of models, for example those obtained by NMR. The current Tcl/Tk-based version of the program is available at http://www-ibmc.u-strasbg.fr/arn or by request from sacha@igbmc.fr. These tools will be available in a future release of the *PHENIX* software.
